# Champing at the Bit for Improvements: A Review of Equine Welfare in Equestrian Sports in the United Kingdom

**DOI:** 10.3390/ani12091186

**Published:** 2022-05-05

**Authors:** Tim Q. Holmes, Ashleigh F. Brown

**Affiliations:** 1 Independent Researcher, Bideford EX39, UK; 2 Independent Researcher, Glasgow G82, UK; ashleighfionabrown@gmail.com

**Keywords:** equine welfare, equestrian sports, social processes, institutions, improving animal welfare

## Abstract

**Simple Summary:**

This review explores the evidence for the effect of equestrian sports on equine welfare and highlights scenarios in which their welfare can be impaired and where efforts are being made to improve it. We discuss animal welfare as a complex and disputed issue, clarifying what it means and how it can be measured. We review many aspects of animal welfare risk to the life of equids used for sport from foal to retirement. This is followed by a unique analysis of the institutions and social processes influencing equine welfare, exposing the challenges faced by a broad range of stakeholders with differing ethics, attitudes and values. We conclude with recommendations to ensure good welfare for all equids used for sport in the UK and beyond.

**Abstract:**

Equestrian sports, including racing (e.g., flat, steeple-chasing, harness or donkey derby); show-jumping; cross-country; dressage; polo; polocrosse; endurance; carriage driving; vaulting and hunting; are hugely popular in the UK, and they involve a significant number of people, both as participants and spectators, and tens of thousands of equids. In this paper, we discuss animal welfare as a complex and disputed issue, clarifying what the term means and how it can be measured. We review many aspects of welfare risk to equids used for sport, addressing issues encountered throughout their lives, including housing, feeding, veterinary intervention, shoeing, handling, training, breeding and equipment. This is followed by a unique exploration of the institutions and social processes influencing equine welfare. The institutional components comprise the rules of competition, the equids, attributes of the stakeholders and the space where participants strive to achieve a common purpose. We endeavour to untangle the most significant elements that create barriers or provide opportunities for equine welfare improvement. We expose the challenges faced by a broad range of stakeholders with differing ethics, attitudes and values. Evidently, there are many welfare risks to which equids used in sports continue to be exposed. It is also evident that significant improvements have occurred in recent times, but there remains a barrier to reducing the risks to an acceptable level. We conclude with recommendations regarding a process for change, involvement of stakeholders and management of knowledge to improve equine welfare that involves identifying and prioritising the risk factors and ultimately leading to interventions, further research and/or education.

## 1. Introduction

There are currently an estimated 1,519,447 horses and 27,592 donkeys living in the United Kingdom (UK) [1]. Although some are companion animals or pets, and others support livelihoods through direct income generation (such as tourism or riding schools), the majority are used for leisure and sporting activities [2]. Sports involving horses, donkeys and their hybrids (equids, subsequently) in pursuit of competition, entertainment or athletic excellence are diverse and include the following: racing (e.g., flat, steeple-chasing harness or ‘donkey derby’); show-jumping; cross-country; dressage; polo; polocrosse; endurance; carriage driving; vaulting and hunting. This diversity in activities, training and management regimens, tack/equipment and demands upon the animals—coupled with the expectations, variation in skills and pressures upon the people responsible for them—create a broad range of scenarios and issues that can influence the welfare of equids used in sport.

The goal of this review is to present a summary and critical assessment of the literature, providing an overview of the key effects on equids used for sports, i.e., a narrative integrative review. The focus has been on peer-reviewed evidence wherever possible, but when this has been lacking, other sources of evidence have been sought, including published books, websites and newspaper articles. We acknowledge the potential for the increased likelihood of biases in non-peer-reviewed sources; nevertheless, they can be useful to enrich the discussion. When citing non-peer-reviewed sources, efforts have been made to ensure sources are as reliable as possible by only using works by professionals (e.g., academics), professional organisations with expertise in the relevant subject matter (such as the International Society for Equitation Science) and non-governmental organisations (NGOs) operating in this field (e.g., equine welfare charities).

Throughout this review, for simplicity, the term ‘equestrian sports’ is used in its broadest sense, i.e., to refer to all sports involving the riding/driving of horses, and does not exclude any specific disciplines (e.g., racing).

The focus of this review is the use of equids in competitive sporting activities, both professional and amateur, in the UK, with particular consideration of the welfare implications associated with breeding, management/husbandry, training and post-competition. In addition, this review explores the institutions and social processes influencing equine welfare and concludes with recommendations to ensure good welfare for all equids used for sport in the UK.

### 1.1. History of Horses Used for Sport in Britain

Horses have a long history of being used in Britain for riding, traction and draught, with evidence of bridles from the Bronze Age (3100–300 BCE) and bit-related damage in the mouths of horses from the Iron Age (500–332 BCE) when metal bits were initially introduced [3,4]. The first documented use of horses for sport dates back to 800 BCE when chariot races took place, and in subsequent centuries, the Romans raced horses during high-profile events in purpose-built arenas [5]. At the end of the 11th century, horses began being used for sport in Britain when flat racing commenced in England [5]. The first references to formal racing with horses in Britain go back to the 16th century, and the purposive breeding of race-horses can be traced to the rule of King Charles II of England in the 17th century [6], with the Jockey Club founded in England in 1750 [5]. Subsequently, the popularity of racing grew, both for participants and spectators, aided by its commercialisation and betting [7]. According to Huggins [8], between the two world wars, horse-racing was amongst the largest and most sophisticated of the leading British industries and the leading sport, if betting is considered (cf. cricket for participation and football in terms of spectatorship), with the year-round season bringing in large crowds. Equestrian jumping subsequently emerged in Britain towards the end of the 19th century, and the first major show-jumping competition was held in 1907. Then, in 1923, the British Show Jumping Association was founded and survives to this day as British Showjumping [9]. The history of dressage in Britain has its roots in the mid-17th century, but it was not until 1955 that the first national dressage championships were held [10]. Although eventing featured as a sport on the international stage at the Olympics, competitive eventing was held for the first time in England in the 1940s, providing opportunity for British riders to train for future international competitions [11]. Polo originated in Persia (Iran) in 600 BCE and was first played in the UK in 1869, with the first UK polo club being established in 1872 [12]. In 1898, the County Polo Association (CPA) was established to look after the interests of polo clubs [12]. The Hurlingham Polo Association took over the role of the CPA in 1949 and is currently the governing body for polo in the UK [12].

### 1.2. Scope and Scale of Equestrian Sports

To provide insight into the scope and scale of equestrian sports in the UK, an overview of participation in the most popular equestrian sports is summarised below, based on the information publicly available. Where possible, data relating to the UK has been used, but it should be noted that some sources refer to Great Britain and not the UK. These data are not exhaustive or cumulative, as, in some cases, the same animals/riders may participate in more than one equestrian discipline. There is also cross-over between professional and amateur sports, as some amateur competitors may still earn prize money, and some high-level riders may not have this as their exclusive source of livelihood.

#### 1.2.1. Horse-Racing

In 2019, a recorded 23,537 horses were in training for flat and jump racing at the 60 existing race courses where 1444 fixtures were held in Great Britain [13]. The number of runners in total were 91,397, and the total race values were £161,064,000 [13]. The same year saw a turnover of on-course and off-course horse-race betting in Britain of £227.5 M and £4.2 BN, respectively [14]. It is estimated that 7000 horses leave British racing each year: some are retired to stud for breeding, some are transported abroad to race, some are used in point-to-pointing and the rest (over 40%) are retrained for alternative equestrian disciplines or recreational activity ([15]; see Section 3.3.2 and Section 3.4).

#### 1.2.2. Show-Jumping

British Showjumping, the governing body, in 2018 had 16,349 members and held 2530 shows with 18,203 equids [16]. In addition, there are other membership organisations that hold events throughout the country, such as Showjumping UK and Horse Events [17,18].

#### 1.2.3. Eventing

Annually, over 170 one- or three-day events are typically scheduled throughout Britain, with over 94,000 entries, and the governing body, British Eventing, has 15,000 members [19].

#### 1.2.4. Dressage

In 2018, the governing body, British Dressage, had more than 17,000 members, with 23,577 horse registrations and nearly 2886 competition days [20]. Another membership organisation, Dressage UK, also organises events and competitions throughout the country [21].

#### 1.2.5. Polo

Today England ranks among the three biggest polo-playing nations, together with Argentina and the United States of America [22]. The Hurlingham Polo Association, consisting of 59 outdoor clubs in the UK and 32 overseas clubs and associations, is responsible for the regulations and rules under which the game is played [12,22].

Overall, it is estimated that 27 million people in Britain have an interest in the equestrian sector, which has an economic value of £4.7 billion [23]. The statistics summarised above indicate the popularity of equestrian sports and the involvement of a significant number of people, both as participants and spectators, and equids. The equestrian sports industry plays an important role commercially, with successful horses at the elite level earning large amounts of prize money and prestige for owners, trainers and riders/drivers, and after their competitive careers, some horses can become valuable breeding assets.

An estimated 85,000 people are employed in the equestrian sports sector in the UK and Ireland [24]. Their roles may include riders, trainers, breeders, grooms, stable-hands, course staff, farriers, veterinarians, vendors of tack and other equestrian goods, sports journalists, event professionals and photographers. In relation to racing specifically, betting is another significant commercial facet of the industry, employing bookmakers, betting-shop staff and engaging professional gamblers. Huge sums of money change hands, including taxes paid to the government, providing financial incentives to support the industry [25]. In addition to paid work and commercial activity, there is also a considerable element of voluntary activity within the equestrian sector [26].

### 1.3. Equine Breeding

Natural selection has rendered equine species with speed, strength, stamina, alertness and cooperation [27], enabling them to survive and thrive as prey species in the habitats for which they evolved—generally considered to be the plains of North America for horses [28] and arid lands of northern Africa for donkeys [29]. Subsequent selective breeding has capitalised on some of these traits, as well as equids’ adaptability for different purposes, through breeding to enhance characteristics that are particularly desirable to humans. This includes physical traits such as variations in size, strength, conformation and athleticism and psychological traits such as temperament, learning ability and pliability to meet different human needs or wants in the context of domestication [30]. Despite many centuries of close affiliation with humans, modern-day domesticated equids appear to have retained much of their natural behaviour repertoire. Many of the behaviours reported in feral (e.g., Camargue horses and Exmoor ponies) and wild equine species (Przewalski’s horse, three species of zebra and three species of wild ass) can be similarly observable in domesticated equids; for example, vigilance and social behaviours such as allo-grooming, play and pair-bonding [31]. Although herd dynamics may alter in free-living equids according to geographical constraints and resource pressures [32], studying these populations has provided important information about natural behavioural time budgets and highly motivated behaviours in equine species [33].

The modern-day results of centuries of selective breeding are eighteen ‘British native’ breeds [34], adapted for the requisite work and living conditions at the time, which include large and powerful Shire and Clydesdale horses bred for heavy draught work; flashy, gaited Hackneys for eye-catching carriages and small, hardy Shetland and Eriskay ponies adapted to the harsh climate of Scotland’s northern islands [35]. Today, there are 47 equine breeds present in the UK [34], and British native breeds are widely intermixed with international breeds and bloodlines. The hybridisation of horses and donkeys to produce mules and hinnies also occurs on a much smaller scale.

## 2. Equine Welfare and Assessment

### 2.1. Defining Animal Welfare

In order to assess it meaningfully, it is important to consider what is meant by ‘animal welfare’, and there are varied definitions of the term (see, for example, [36,37,38,39,40,41]). These include ideas that welfare is the animal’s state regarding attempts to cope with his/her environment [42], incorporating both physical health and mental wellbeing, which are influenced by factors such as those from the ‘five freedoms’ [43] and the ‘five domains’ [44], and concepts focusing on the quality of life, such as ‘a life worth living’ [45]. Differences in definitions may arise due to differences in moral or ethical standards of society [46] and stakeholders being inclined to emphasise different aspects of animal welfare [47] (e.g., health, productivity, behaviour, ‘naturalness’, etc.). Additionally, welfare assessment methodologies may focus on different types of measures, such as resource inputs, environmental conditions or animal-based indicators [48]. Indicators within these groups can also differ; for example, animal-based indicators might be physiological (e.g., hormone levels or heart rate variability), behavioural (e.g., free choice or approach tests) or based on physical characteristics (e.g., skin lesions or gait). The increasingly widespread desire to focus on animals’ subjective experiences (i.e., the way animals consciously experience pain, pleasure, suffering and a range of emotions [49]) can be complicated further by both the influence of the human-animal relationship and humans not always being able to reliably or correctly interpret the experiences and feelings of non-human animals [50].

Three areas of commonality within contemporary definitions of the term ‘animal welfare’ relate to: (i) an animal’s feelings or emotions, (e.g., the animal being happy or suffering from pain or another negative affective state); (ii) an animal’s health and ability to function biologically; and (iii) an animal’s ability to perform a normal array of natural behaviours [47]. This latter point, regarding normal behaviours, presents a challenge when applying to equids who have been domesticated for many generations and selectively bred for human-directed work activities and to adapt to unnatural interspecific relationships, i.e., the closely entwined lives of domesticated equids and humans. Whilst, as previously mentioned, equids have retained many behaviours of their wild ancestors, they may have different behavioural needs in a captive environment. Additionally, some natural behaviours may be detrimental to welfare (e.g., fighting or fleeing predation), and conversely, some unnatural behaviours may be beneficial to welfare (e.g., positive human–animal interactions such as grooming or stroking).

There is also evidence that the ways in which stakeholders understand the concept of welfare might act as a barrier to the improvement of some equine welfare problems, highlighting the importance of the consideration of stakeholder constructs [51] to facilitate improvement in equid welfare.

### 2.2. Measuring Equine Welfare

As with welfare assessments of other species, a significant challenge in measuring equine welfare is being able to confidently and reliably interpret the animals’ subjective experiences. It is challenging enough for human doctors to treat patients who can explain the location and intensity of their pain or psychologists who can ask patients about their mental state. Accordingly, for investigating the welfare of non-verbal animals, there is an important role of visible indicators that reflect an individual’s subjective perception of their situation. These indicators must also be scientifically robust and validated to enable the most accurate interpretation of an animal’s welfare status based on the current evidence.

Reliable indicators of horse welfare (i.e., those with a good evidence base) were reviewed by Lesimple [50]. Key examples from this review include the following:

- Health-related—body lesions; body condition score and specific postures (lameness, prolapse, hoof condition and cough/discharges);
- Postural—ear position and neck shape;
- Physiological—cortisol (faecal, blood, hair and saliva); serotonin/oxytocin; white cell count and heart rate/heart rate variability;
- Behavioural—behavioural repertoire; time budgets; reactions to humans and cognitive bias (yawning, play, attentional state and vacuum chewing);
- Acoustic—snort.

In addition to developing a list of robust indicators, a practical process to collect and interpret data and to implement change is critical to ensuring that welfare measurements lead to welfare improvements. Endeavouring to go beyond the intention of providing good animal welfare to establishing working rules, the ‘five freedoms’ framework that identified sources of poor welfare was developed in 1993 [52]. All four nations of the UK have adapted the ‘five freedoms’ in their animal welfare acts (i.e., *Animal Welfare Act 2006 (England and Wales)*, *Animal Health and Welfare (Scotland) Act 2006* and *Welfare of Animals (Northern Ireland) Act 2011*), which all state that “an animal’s needs shall be taken to include:

- its need for a suitable environment;
- its need for a suitable diet;
- its need to be able to exhibit normal behaviour patterns;
- any need it has to be housed with, or apart from, other animals;
- its need to be protected from pain, suffering, injury and disease”.

Whilst these acts aim to avoid conditions that may lead to poor welfare and to promote positive welfare, a limitation of the ‘five freedoms’ approach is that the framework itself is not explicit about what level of welfare is acceptable or optimal, nor does it address alternative needs of different animal species. For example, it may not be feasible to provide a ‘suitable environment’ without pre-existing knowledge of an equid’s natural habitat and associated physical and behavioural needs.

A more detailed and prescriptive method for assessing animal welfare is the ‘five domains’ model [44], which focuses on the following domains:

- Nutrition
- Environment
- Health
- Behaviour
- Mental state

This model has been refined over the years following its initial introduction by Mellor and Beausoleil [53,54] and adapted for assessing horse welfare [55]. The first four domains focus on factors that give rise to specific negative or positive subjective experiences, which, in turn, contribute to the animal’s mental state, as evaluated in the fifth domain. The ways in which the domains interact with each other is also considered, with the aim of providing an overall assessment of whether the animal’s welfare experience is satisfactory or not. The current model also includes guidance on how to evaluate the negative and/or positive impacts of human behaviours (including those of owners, trainers, riders, handlers, farriers and vets) on animal welfare and the importance of seeking not only to remove harms but also to provide opportunity for positive welfare experiences [56].

The application of the ‘five domains’ model for assessing horse welfare focused on the following interventions: weaning, diet, housing, foundation training, ill health and veterinary medical and surgical interventions, elective procedures, care procedures, restraint for management procedures, road transport, activity—competition, activity—work, activity—breeding females and activity—breeding males [55]. McGreevy and colleagues [55] also identified the importance of including structured reviews of the relevant literature for each component to ensure that understanding goes beyond the backgrounds, experiences and biases of the people involved in the welfare assessment, as well as flexibility, for example, to address individual differences of equids, such as previous learning, temperament and personal preferences.

Once any welfare problems have been clearly identified, there is then the opportunity to conduct an assessment of animal welfare risk, where risk is defined as “a function of the probability of negative welfare consequences and the magnitude of those consequences, following exposure to a particular factor or exposure scenario, in a given population” [57]. One example of this process, developed by the European Food Safety Authority [43], incorporates (a) a scenario in which an animal is exposed to a welfare risk, (b) evaluations of the nature of animal welfare effects associated with that risk and (c) calculations (including attendant uncertainties) of the probability of the occurrence and magnitude of the risk. Other considerations regarding welfare assessment include severity, i.e., the product of the intensity and duration of harm [58,59,60] to equids. Assessments regarding severity should consider both physiological and behavioural parameters [61].

Fundamentally, a comprehensive formal process for improving equid welfare requires a holistic, objective, quantifiable, unambiguous evidence-based approach that identifies the risks for negative welfare and opportunities for positive welfare, prioritises them and then implements welfare improvement interventions and appraises their success of welfare improvement activities [62,63,64]. Achieving such a process that is effective is, undoubtedly, hugely challenging, primarily due to the complexities, scientific debate around definitions and methods, ownership of processes, logistics and costs. Many of the issues relate to institutions and social processes are discussed in more detail in Section 4.

### 2.3. Making Decisions with Incomplete Knowledge

Even experts might have varying opinions on equine welfare because of differences in individual beliefs, values, experiences and interpretations of incomplete information. To address this issue, the structured elicitation of expert judgement using the Delphi method can be useful to obtain a consensus and informed decision-making [65,66]. This process can reduce bias and mitigate error among experts; deal with challenges including overconfidence, anchoring to available data and definitional ambiguity and is a popular method for assessing expert opinions on a topic [67,68]. It has been used in the UK for assessing the welfare of farm animals, cats, rabbits and horses [69], in the case of the latter, employed to prioritise the most important welfare issues in horses and those that warrant further research efforts or owner education.

The ‘precautionary principle’ is another important consideration when dealing with incomplete knowledge. This principle arose to guide decision-makers when considering the likely harmful effects of their activities on human health and the environment when exposed to uncertain risks, i.e., taking preventive action in the face of uncertainty [70]. It has since been modified for application in questions of animal sentience by addressing serious, negative animal welfare outcomes.

Birch [71] adopted two ideas from John [72] that help with interpretation:

- “An epistemic rule (a rule about the burden of proof): when there is a live scientific hypothesis that posits a causal relationship between human action and a seriously bad outcome, we should set an intentionally low evidential bar for the acceptance of that hypothesis in the context of formulating policy.”
- “A decision rule (a rule about action): once we have sufficient evidence of a threat of a seriously bad outcome, we should act, in a timely and cost-effective manner, to prevent that outcome.”

There is also acknowledgement that waiting for overwhelming evidence to be generated and a clear consensus to be attained can be costly, and there is often a need for prompt responses to serious developing threats. The slow pace of the progression of science and policies presents a challenge in this regard.

Birch developed the following principle to be adopted when considering animal sentience:

- “Where there are threats of serious, negative animal welfare outcomes, lack of full scientific certainty as to the sentience of the animals in question shall not be used as a reason for postponing cost-effective measures to prevent those outcomes.”

With this in mind, he proposed two rules for consideration when dealing with animal sentience:

- “For the purposes of formulating animal protection legislation, there is sufficient evidence that animals of a particular order are sentient if there is statistically significant evidence, obtained by experiments that meet normal scientific standards, of the presence of at least one credible indicator of sentience in at least one species of that order.”
- “We should aim to include within the scope of animal protection legislation all animals for which the evidence of sentience is sufficient, according to the standard of sufficiency outlined in the first rule.”

It is apparent that this framework, based on the ‘precautionary principle’, could be readily adapted for equids used in sports.

## 3. Animal Welfare Risks

There are welfare-influencing factors that are relevant throughout the equine athlete’s lifetime, particularly those pertaining to husbandry, handling and veterinary interventions. Additionally, there are some that are more pertinent before, during or after their competitive career.

### 3.1. Life Stage: Throughout Lifetime

The domestication of equids and the proximity to which they are forced to live with humans can present fundamental welfare risks by disrupting equids’ ability to live in the natural conditions and social groupings for which they are adapted. Although the domestication process might have led to the development of some behaviours that are beneficial to equids, such as tameness (lack of fear), there remain many scenarios where equids do not appear to benefit. For example, equids, who are highly social animals, in a domesticated context are often prevented from living in natural herd structures, which are important to enable developmental and affiliative behaviours (e.g., social learning, play, establishment of stable rankings within the herd and shared vigilance) [73]. For many domesticated equids, compromises are constantly sought between optimal conditions for positive welfare and the practicalities of husbandry and training, which are often dictated by economical and spatial constraints, and convenience for humans.

As prey species, equids are highly motivated to take flight (i.e., run away) when faced with potentially dangerous stimuli. Many equestrian sports routinely demand this flight response, which may be caused by a fear reaction or performed in response to a conditioned cue from the rider/driver, and this high state of arousal can be difficult to control [74]. Additionally, the strongly motivated ‘flight, fight and freeze’ behaviours are often inhibited by accommodation, restraint or demands to proceed towards a perceived threat, usually for purposes of management convenience, the protection of both humans and equids or occurring in the course of sport participation. Human injury rates in equestrian sports are high in comparison with other sports [75], so control of the horse is of paramount importance for the safety of both rider and animal [74].

#### 3.1.1. Housing

Despite being contrary to natural living conditions, individual stabling, often for a large proportion of the daily time budget, remains the dominant method of housing equids involved in sports [76]. Whilst this affords more carefully controlled and convenient management of the animals, this norm risks exposing equids to behavioural inhibition, under-stimulation and frustration [77].

Herding behaviour is important as an innate survival mechanism; therefore, proximity to, and company of, conspecifics is essential for good welfare. Yet, equids who are housed singly in stables are often prevented from tactile (and, sometimes, also visual and auditory) interactions with conspecifics and agency over contact with individual equids [78]. Conversely, equids may live in forced proximity to conspecifics they would not choose should they have the agency to select which individuals to associate with or avoid. The freedom to utilise avoidance behaviours to avert conflict can reduce agonistic interactions, social stress and susceptibility to disease and promote a harmonious social life [79]. Confinement or restraint restricts kinetic behaviour, particularly locomotion, and limits the opportunity for exploration, which equids show high motivation to perform [80]. The inability to perform highly motivated natural behaviours often results in stereotypies and other abnormal behaviours [81,82]. For example, a thwarted motivation for roaming in confined equids can manifest in abnormal locomotory behaviours such as box-walking and weaving [73,83].

Poor air quality (e.g., due to low air turnover, high levels of dust or ammonia) in some indoor stabling environments can contribute to respiratory problems [84,85], and some hoof problems can be created or exacerbated by prolonged periods of restricted movement (e.g., thrush in stabled equids) [86,87]. The inhibition of natural levels of locomotion and the associated hoof capsule expansion, contraction and blood circulation that would occur in free-ranging equids and, also, potentially unhygienic underfoot conditions if standing in soiled or damp bedding are contributing factors to hoof problems [88].

#### 3.1.2. Feeding

Equids are physically and behaviourally adapted to eat little and often and would naturally spend a majority of their time budget grazing—typically ~60% but more in times of food scarcity [89,90]; therefore, they remain highly motivated to replicate these feeding patterns in a domesticated setting [91]. Feeding regimens typical of equine athletes usually involve a higher proportion of concentrates to roughage and limited feeding and grazing times [76,92,93]. These unnatural feeding practices, especially a diet low in roughage and high in concentrates, can manifest physically in gastric ulceration [94,95] colic and choke and behaviourally in oral stereotypies due to inadequate time spent grazing or chewing [92]. Thwarted motivation for ingestive behaviours—particularly feed-seeking, mastication and swallowing—underpins many behavioural problems that manifest in captive equids exposed to unnatural feeding regimes, such as the development of oral stereotypies (e.g., crib-biting or wind-sucking) [92,96]. However, there are opportunities even for sporting equids who require additional nourishment to meet the demands of competition, to enrich feeding to replicate natural feeding patterns more closely, such as providing a greater grazing opportunity, sufficient roughage available over a long period of time and a reduced dependency on meeting calorific requirements through concentrated feedstuffs that are rapidly consumed [76,92].

#### 3.1.3. Veterinary Intervention

There are several veterinary interventions that may occur throughout a sports equid’s life that could be considered unnecessary for the animal’s health, occurring solely for performance reasons. These vary according to the nature and demands of the particular sport but appear from the available literature to be more prolific amongst horses involved in racing compared to other sports. Although the British Horseracing Authority phased out the practice of ‘tubing’ (tracheostomy procedure) from racing in the UK as of 2012 [97], ‘wind operations’—which aim to improve the flow of air into, and carbon dioxide out of, the lungs (e.g., Hobday, tie-back or tie-forward procedures (ventriculocordectomy, laryngoplasty and laryngeal advancement, respectively))—remain common to the extent that, as of 2018, in Britain horses racing for the first time after wind operations must be formally declared [98]. The administration of drugs, including banned performance-enhancing substances (e.g., anabolic steroids) by injection or alkalising agents (known as ‘milkshakes’) via a nasogastric tube, occur within racing [99]. For horses undergoing these unnecessary invasive or painful procedures, there is a risk of psychological distress and discomfort, in addition to that caused by any restraint. There is also the risk of physical harm, including oesophageal tears and aspiration pneumonia from nasogastric intubation [100,101] and abscess or toxicity from injectables [99].

Intraarticular (joint) injections and regenerative therapies such as stem cell treatments are utilised to improve or restore performances in horses involved in various sporting disciplines [102]. Iatrogenic complications include septic arthritis, tissue contamination or the development of aberrant cells [103,104,105,106]. Whilst some of these may be performed as remedial therapies to reduce the animal’s discomfort from injury, sometimes they might be performed primarily for performance improvement or to enable a return to competition rather than optimisation of the animal’s welfare. The literature indicates a lack of standardised, evidence-based treatment protocols for some such procedures, suggesting that the best practice has yet to be established [107,108,109].

#### 3.1.4. Hoof Management

In the UK, 96% of horses receive hoof care from a farrier every 6–8 weeks [110], and farriery standards are highly regulated, with quality assurance provided by the Worshipful Company of Farriers [111]. In light of the welfare risks involved in farriery and the level of knowledge and skill required to mitigate these, to ensure equid safety, it is a requirement that farriers are fully trained (typically via apprenticeship over four years) and registered in order to practice legally.

Whilst the shoeing of equids is commonplace and normalised, and for some sports it is mandatory, e.g., flat turf racing [112], this is an inherently unnatural process. A trend towards barefoot (unshod) management has been seen in the UK over recent years [113]. However, shoeing is still prevalent for reasons of hoof protection, improved traction or correction of abnormality and is often required to counterbalance the implications of the physical demands upon sporting equids. For example, shoeing can aid sporting performance and safety (e.g., use of studs for race-horses to provide traction) or prevent damage to the hooves (e.g., protect against excessive wearing down of hoof wall due to working on abrasive ground surfaces). Despite the careful regulation of the farriery profession and a high level of service-seeking by equid owners, problems with the hooves are still the most frequent owner-reported cause of lameness in horses [110], and in a survey undertaken to explore client–farrier relationships, 89% of client respondents reported having encountered hoof problems in the previous five years [114]. This appears to indicate that either the physical demands being placed upon the equids or some aspect of their husbandry and management may be creating a challenge in terms of the ability of the feet to withstand damage. Some challenges in the client–farrier relationship have also been identified: 41% of respondents experienced difficulties finding a farrier they trusted, 23% had a criticism of their farrier and 29% felt their farrier would have criticisms of their demeanour [114]. This dynamic could potentially be a contributing factor to the persistence of hoof problems, for example, if clients fail to follow farriery advice due to a lack of trust or if criticism of a farrier created a scenario in which s/he had to adapt their practice to meet a client’s preference.

The properties of shoes influence equine biomechanics, and if farriery is not performed to a very high standard the repeated shoeing and nailing of hooves can risk disrupting normal hoof function (e.g., contracted heels due to poor hoof capsule expansion) or causing an iatrogenic injury (e.g., nail prick) [115,116]. The farriery process may also be uncomfortable, unpleasant or stressful for equids, particularly if handling is poor.

#### 3.1.5. Handling and Training

The manner of the human-animal interaction can considerably improve or diminish an equid’s welfare experience, and there is strong evidence of the impact of stockpersons’ behaviours on welfare outcomes for various animal species [117]. Considering the duration and frequency of the daily interactions between humans and intensively managed equids used for sport, there is considerable scope for fear, negative anticipation and associated distress if handling is poor and scope for enrichment and positive welfare when the handling is compassionate. Reducing stress responses enables better learning—an important consideration for equids in training—and facilitates the diversion of physical resources towards maintaining health and fitness as opposed to mediating stress responses [77].

The preparatory training of equine athletes is a key component of equestrian sports, and each sport has a suite of ethological challenges, as well as learning and cognitive challenges. Negative reinforcement (i.e., rewarding desired behaviour by the removal of an aversive stimulus) is commonplace as a learning mechanism in training for equestrian sports, as aids, such as pressure on reins or a rider’s leg against the horse’s ribs, are used to instruct the horse—important for both performance and safety [118]. Ethical equitation demands that horses should be trained to respond to minimal pressures [119], although, in some scenarios, the requirement that human safety takes precedence may potentially raise the ‘minimal pressures’ deemed appropriate. Conversely, it is recognised that there are also scenarios in which the equid’s safety will be prioritised and individuals who would incur injury to themselves rather than allow one to occur to their equid.

Equids naturally tend to avoid rather than seek out conflict; hence, an approach by an aggressive individual usually elicits escape or avoidance behaviour [73]. However, in domesticated and training contexts, escape or avoidance is often not feasible for the animal due to restraint (e.g., by the tack) or confinement (e.g., in a manège). Therefore, basing the training of equids on a dominance concept may be detrimental to their welfare, as it creates a scenario in which they cannot exercise their natural coping mechanisms in response to a potential stressor (e.g., avoidance of an aversive stimulus in the form of a trainer affecting a ‘dominant’ position, whom the equid perceives as a potential source of conflict). There are, unfortunately, examples of riders/drivers, trainers and handlers who—perhaps believing they have to place themselves in the dominant ‘alpha position’ in relation to the horse due to a misunderstanding of equid behaviour or learning theory or motivated by their own fear—resort to harsh or inhumane training practices that cause the animal fear as a means of achieving ‘submission’ [120].

Some training and events can be too arduous for horses to undertake safely, such as those involving excessive distances or demanding obstacles, as evidenced by injuries and fatalities [121,122]. National hunt racing, for example, requires horses to jump over multiple fences and ditches combined with a relatively long distance and includes the Cheltenham Gold Cup and the Grand National at Aintree, at which there are regularly significant numbers of injuries and deaths [123]. Intensive physical training of immature equids, especially when they must frequently undertake a prolonged fast pace or weight-bearing work, can cause acute and chronic musculoskeletal injury and damage (e.g., bucked shins) [124,125]. In race-horses, the risk of musculoskeletal injury was found to be associated with training at speed over long distances and increasing the frequency of fast-paced training days [126].

Learned helplessness can occur as an outcome of training when an animal is presented with aversive stimuli that cannot be reliably controlled and are too challenging to habituate to [77]. This results in a psychological condition when animals learn that they have no control over unpleasant or harmful conditions, actions are futile and they are therefore helpless to ameliorate the negative experience [127]. The consequences of learned helplessness include reduced motivation, anhedonia and cognitive deficits [127]; therefore, this state has negative implications for both equine welfare and sporting performance.

Anthropomorphism can also cause problems during training if trainers perceive that horses have some voluntary involvement and are motivated by benevolence or malevolence and, thus, held responsible for their behaviour (for more on anthropomorphism, see Section 4.4). A common example is the use of the word ‘vice’ to describe resistance or evasion behaviours [77], a word defined as “a moral fault or weakness in someone’s character” in relation to humans [128]. One of the contributary factors to this is a lack of consistent descriptive terminology within the equestrian sector [129].

Trainers, riders/drivers and handlers must aim to establish a positive and compassionate relationship with their horses based on the use of clear, consistent and timely cues in order to optimise their welfare during training. They should be aware of natural equine behaviours and functions in the context of social organisation and of the possible repercussions of describing their training processes and interactions with horses.

The specific manner of training young horses will vary according to the experience, attitude and preferences of the individual trainer—there is not at present any one standard qualification in equine training, nor requirement to register as a trainer (in contrast to the rigorous quality assurance of the farriery profession, for example, as previously mentioned). Racing is an exception, as trainers require a licence from the British Racing Authority in order to enter horses for steeple-chase, hurdle or national hunt races [130]. Training opportunities in the UK include certificates in ‘horsemanship’ and coaching offered by the British Horse Society [131] and education in ‘natural horsemanship’ offered by private practitioners (e.g., the ‘Join Up’ protocol devised by Monty Roberts which is delivered in the UK by a network of certified trainers). Some trainers also learn via mentoring, apprenticeships and on-the-job learning by working alongside more experienced practitioners, and in some cases, apprenticeships may be supported by vocational training qualifications (e.g., City and Guilds provides formal assessment of equine grooms in racing, breeding and riding and qualifications in horse care [132]).

Whilst conventional training methods have historically been based on the ideas of trainers asserting ‘dominance’ over equids and using predominantly punishment-based techniques, as the evidence base and scientific understanding of equine behaviour and learning theory has grown, a shift towards more ‘sympathetic’ and rewards-based techniques has been emergent [133,134]. The training of equids still often relies on the use of aversive stimuli as a component of classical and operant conditioning methods [135], for example, the application of pressure from the rider’s leg onto the equid’s ribcage to encourage a particular response (i.e., ‘positive punishment’) and removal of the pressure once the desired response occurs (i.e., ‘negative reinforcement’), and are generally considered acceptable in terms of welfare when the aversive stimuli are mild. However, methods causing pain and fear have been challenged, and the role of pain in inhibiting learning and performance (in addition to compromising the equid’s welfare) has also been acknowledged [120,136,137].

### 3.2. Life Stage: Prior to Participation in Competitive Sports

#### 3.2.1. Breeding

There is currently a dearth of evidence on the nature and prevalence of welfare issues and effects of equine breeding, and there are calls within the animal welfare and veterinary professions for this to be addressed [138]. However, the current understanding of animal welfare science, applied ethology and the behavioural needs of equids provide valuable insight into the aspects of breeding practice that have the potential to compromise their welfare and are also useful in guiding consideration of how the equids’ welfare experience could potentially be improved.

The breeding of horses used for sports mostly occurs either (a) ‘naturally’ with the stallion being led by a human to the mare who is also restrained or (b) using artificial reproduction techniques; both of these appear to have a scope for welfare compromise. ‘Natural’ mating typically involves restraining the mare using a head collar or bridle to prevent her from moving away from the stallion and thus avoiding successful covering (mating). The addition of a nose twitch might occur to further reduce fractious behaviour when it is considered that this may pose a risk of injury to the animals or handlers involved. In some cases, the mare’s limbs are hobbled or boots applied to the hind limbs to reduce the risk of injuring the stallion or discouraging him from a successful covering should she attempt to kick him. Similarly, the stallion might be muzzled to prevent him from biting the mare. Whilst it is understood that the rationale for these interventions is to improve safety and reproductive success, they also inhibit the expression of defensive behaviours that are intrinsic to equids’ survival as prey species and, therefore, are likely to be highly motivated or to create psychological distress if they are thwarted. For example, a mare attempting to display a kick threat (or kick with intent) at an approaching stallion may be indicative of her fear of him or of the handling situation more broadly. Her first defensive mechanism of avoidance is removed by the head restraint, and if hobbled, her second defensive mechanism of aggression is also truncated. The thwarting of highly motivated behaviours and experiencing fear have been associated with animal welfare compromises [60,139,140].

In addition to the examples above, Campbell and Sandøe [138], acknowledging the lack of research, identified the following potential welfare risks associated with natural and artificial breeding methods:

Natural

- Transportation: Distress related to the long-distance transport of breeding stallions (see Section 3.3.3 for more information).
- Frequency of cover: Excessive frequency of covering by the same individuals (artificial insemination can alleviate many of the welfare risks associated with this).

Artificial

- Artificial insemination: Anecdotally likely to be minimally painful/stressful for most mares and the attenuation of normal reproductive behaviours for stallions.
- Embryo transfer: Increased need for invasive examination and pharmacological manipulation compared with artificial insemination. Embryo flushing process may be stressful/painful.
- Oocyte retrieval and transfer: Known to be associated with increased heart rate and peripheral cortisol levels and the development of adhesions in other species. No conclusive evidence of long-term welfare effects on foals conceived by oocyte retrieval, although these are known to occur in other species in association with particular uses of culture media.
- Cloning: Increased risk of abnormalities in foals at birth, increased requirement for neonatal intensive care.

It is established knowledge that animals with low genetic diversity, such as those selectively bred for particular traits that are desirable to humans or those breeding from interrelated bloodlines, can be more prone to specific injuries or heritable disorders [141,142]. This is evident in various species, including dogs, cats and primates, as well as equids [142,143,144]. In the case of equine breeds, more than 20% have been identified as susceptible [145], and an overreliance on particular bloodlines or a small pool of the most desirable breeding stock appears to have an increased risk and prevalence of heritable disorders or specific injuries related to conformational defects within some breeds. Examples include lordosis in American Saddlebreds, chronic progressive lymphedema (CPL) in several draught breeds, carpitis in Norwegian trotters or the characteristic flat feet and thin hoof walls and soles in Thoroughbreds (often exacerbated by their training regimens) [146,147,148,149], amongst other conditions. The Thoroughbred is the equine breed predisposed to the highest number of inherited disorders and may be more likely to develop an associated abnormality (e.g., sore or bucked shins and decreased hoof angle) due to the physical stress upon the musculoskeletal system [150,151]. These genetic predispositions are likely exacerbated when susceptible individuals are pushed to their physical limits. The negative effects of selective breeding can manifest in direct welfare concerns for surviving equids affected by symptoms of heritable disease and may also contribute to indirect welfare implications associated with poor performance (such as a greater risk of injury during training or competition) or consequences of ‘wastage’ (e.g., lengthy transportation for export or onwards sale and relocation to multiple different owners). Conversely, selective breeding can also be applied by breeders to intentionally improve breeds’ or species’ resistance to disease or the ability to cope with their environment, which has been explored in cattle, pigs and fishes [152,153,154,155]. Although inbreeding in equine breeds does not always result in heritable defects in all progeny [156], it has been found to negatively affect sporting performance [157]; therefore, there may be both performance and animal welfare benefits to be gained by broadening the genetic diversity amongst equine breeds with high genetic loads and inbreeding depression.

At the population level, there is a surplus of horses intended for professional sport who have proved unsuitable for, or unsuccessful in, competition or obtained career-ending injuries. This is particularly acute amongst racing Thoroughbreds but not exclusively so [158]. Lameness is a key contributing factor to the wastage of equine athletes who may need to be withdrawn from competition or may never reach an elite level on account of musculoskeletal injury, hoof problems and associated lameness [150,159,160,161], and respiratory problems have also been identified as an important cause [158]. Whilst some equids withdrawn from competitive sport may be accepted by charities for retraining and rehoming (e.g., The Racehorse Sanctuary, Heroes and Life After Racing), many others are killed or cannot be traced [162]. There is scarce evidence on the welfare of equids at slaughter in the UK, but reports have indicated illegal practices and serious welfare issues, including killing without prior stunning, protracted deaths and transportation to abattoirs whilst suffering prior injuries [163]. Whilst new regulations ban British race-horses from entering the food chain as of 1 January 2022, this will not address the transportation of race-horses from Ireland for slaughter in British abattoirs [164]. However, at the time of writing, indications are that the British Horseracing Authority is liaising with international jurisdictions and examining European Union legislation to look at the inclusion of all international runners [164].

#### 3.2.2. Brood Mares

An estimated 9% of horse/pony mares in Britain are utilised for breeding purposes [165], either on an informal, small-scale basis by individual owners or commercially as a primary occupation for professional breeders.

Although brood mare welfare has not been well studied, multiple pregnancies and parturition have the potential to incur specific welfare risks, such as recurrent Caslick procedures (vulvoplasty, in which portions of the skin on either side of the vulva are cut off and the open tissues sealed back together with sutures) [166,167]. Caslick procedures are performed to reduce the risk of post-partum uterine infection due to air and bacteria entering the vagina and must be cut open again prior to each foaling, hence the particular concern about brood mares undergoing this surgical procedure repeatedly, which would not be applicable to mares who foal only once or twice in their lives. As elaborated above, there is a risk to mares of pain, discomfort or fear at the time of covering (mating) due to restrictive restraint methods, such as hobbling or nose twitching, and fear of, or injury from, the stallion (e.g., due to the pressure of fore-hooves on the loins or biting the neck). Invasive assisted reproductive technique procedures have the potential to create pain, discomfort, fear or risk of internal injury and infection, and artificial manipulation of oestrus to prolong the breeding season has welfare implications [138]. The equine fertility period can range from 2.5–20 years of age, and brood mares might foal on an annual basis [167].

Weaning, particularly if this occurs abruptly with a sudden separation of the mare and foal rather than via a gradual process, often results in psychological and physiological stress for both foals and brood mares—in some cases, severe enough to result in a loss of physical condition in either party [168,169]. Although research indicates this can be mitigated though gradual weaning programmes [169], these methods are not always implemented, perhaps due to reasons of convenience, limitations of available housing facilities or a desire to wean at a younger age than would naturally occur. Since brood mares are faced with recurrent exposure to weaning stress, there appears to be the risk of both acute and cumulative negative welfare implications of this stressful process throughout their lifetime, although it seems this has not been researched to date.

#### 3.2.3. Stallions

For reasons of management convenience, including the prevention of indiscriminate breeding or concern about stallion aggression and associated injury risks to animals and people, it is common for stallions to be singly housed [170]. This husbandry regime is contrary to the natural living conditions in which they would spend the majority of their lifetime as part of a herd, either as a breeding harem stallion or as a member of a bachelor band [171]. The inhibition of natural behaviours and normal social interactions from unnatural management scenarios can increase psychological distress, stereotypic behaviour and aggression, including self-mutilation [172,173].

There can be a risk of injury to stallions from mares during covering, often managed through the restrictive restraint of mares or use of false mares. Stallions may also experience frustration from the thwarting of highly motivated reproductive behaviours (e.g., social interaction with the mare prior to covering), as these cannot be performed fully during in-hand covering or when artificial reproductive technologies are used. Stallions are also at risk of stress, immunocompromise, disease and exhaustion if they experience long-distance transportation to accommodate the breeding seasonality in different parts of the world and when artificial insemination is not permitted (e.g., Thoroughbred race-horses) [138]. Linked with this, the intensity of breeding schedules for natural coverings can be both physically and psychologically demanding upon the stallion [138].

#### 3.2.4. Foals/Offspring

As mentioned above, weaning is a source of considerable stress for foals, and abrupt weaning methods in particular can result in increased distress vocalisation, locomotion, abnormal behaviour, inappetence and growth rate reduction [168,174]. It has been suggested that the artificial manipulation of breeding seasons may influence a foal’s propensity toward obesity later in life [175]. The transference of genetic weaknesses may lead to a nonviable foetus, abortion, perinatal fatalities or a chronic welfare compromise throughout the lifetime of a viable offspring born with heritable conditions, which cause discomfort or inhibit mobility (e.g., congenital club foot or other limb deformities) [176]. Onwards sale, transit and rehoming can also expose young-stock to pathogens and stress due to transportation, unfamiliar animals and novel surroundings [177,178].

The early training period for foals and young-stock has the scope to provide both opportunities for positive welfare—if the youngster develops positive associations with humans and enjoys pleasant experiences and interactions and positive stimulation—and also welfare risks, particularly if there is an overuse of punitive training methods and excessive cognitive or physical demands at an early age [81,179]. Studies have indicated that very early ‘forced’ handling of foals (in the first days of life) does not appear to bestow important benefits and that handling of the dam may be a more important factor in facilitating positive associations between the foal and humans at that stage [180,181]. Similarly, group-housed young-stock performed better in training and showed less aggression towards trainers than singly housed young-stock [182], suggesting that equid–equid relationships influence equid–human relationships. Therefore, optimising the welfare of foals and young-stock requires careful consideration of the nature of interactions with the dam, timing of handling and the housing method. Evidence in several species indicates that positive or negative associations based on early experiences of learning can persist throughout the animal’s life, with negative experiences increasing the fear behaviour, stress responses and associated negative alterations to the physiology (e.g., immunocompromise) [183,184,185].

### 3.3. Life Stage: During Participation in Competitive Sports

#### 3.3.1. Use of Equipment

Alongside the knowledge, skills and attitudes of trainers, another important determinant of an equid’s experience in training and competition is the tack and equipment used. Tack provides a necessary means of communication with the equid and control over pace, speed, balance and direction, which is an important safety consideration in sports. The equipment used in equestrian sports—such as bits, spurs, nosebands and whips—are often utilized in conjunction with aids from the rider/driver that apply pressure to various parts of the animal’s body to amplify or refine the signal to the equid. Whilst these do not always incur pain or distress, they are still instruments of negative reinforcement or punishment and function through creating an inherently aversive stimulus to which the animal responds [186] and, thus, have the potential to impair welfare (see, for example, [187,188,189,190,191,192,193]). This risk may be increased if equipment is used by people who are inexperienced, uninformed about the manner in which these artificial aids function or lack knowledge of equine behavioural needs; conversely, when users are experienced, knowledgeable and compassionate, negative welfare implications should be minimised.

The Federation Equestre Internationale (FEI; an international governing and non-profit organisation focused on equestrian sports) and national equestrian federations have rules to protect the health and welfare of horses during competitions. However, there is still a need for information relating to the different types of equipment used and the related occurrence of injuries to guide the development of rules to truly protect horses [194].

Bridles (typically comprising reins, cheekpieces, noseband, browband, headpiece and bit) are almost always used by riders/drivers in equestrian sports to control the speed and direction of the horse. Bridles are designed to function by applying pressure to sensitive areas of the horse’s head (e.g., poll, chin and nose) and, when bits are present, in the oral cavity (e.g., bars, tongue and palate) [74]. Spurs and whips are used to encourage the horse to increase speed, increase activity or move in a specific direction. In some cases, tongue-ties are used to prevent horses from placing their tongues over the bit, which can impair bridle functioning and, therefore, reduce control over the animal. However, research has indicated that, for a race-horse working at a gallop, tongue-ties can impair the airway [195,196].

The use of training aids, such as draw reins, side reins, Market Harborough, chambon or de Gogue, also have the potential to cause discomfort; pain (to the mouth, implicated muscles and any pressure points) or behavioural inhibition and thus risk creating fear and distress [192,197], particularly when used excessively, in inexperienced hands or by trainers without detailed knowledge of equitation science. Many of these training aids instigate changes to the animal’s head carriage and neck position and the use of muscles during motion (e.g., in the back or hindquarters); therefore, muscle fatigue and pain can also be implicated [197]. Some training methods do not use restrictive training aids but may use aversive stimuli through intentionally causing pain or unpleasantness to the equids. Examples of aversive practices include raising poles as the animal jumps over them, so they collide with the hooves as a means of encouraging higher clearance over poles or whipping the limbs of a horse with each step to encourage the limb to be lifted higher (personal observation).

A clear understanding of the appropriate and inappropriate uses of training equipment by riders/drivers and trainers is important to minimise the welfare risk. It has been suggested that this is currently lacking [198] and that there is a need for operators to assess whether artificial aids are commensurate with the principles of learning theory and that the continued use of training aids is underpinned by regular and objective assessments of the individual horses’ progress within a training or rehabilitation programme [199]. To promote ethical training, in recognition that the use of aversive stimuli has the potential to negatively impact equine welfare, the International Society for Equitation Science (ISES), a professional membership body of equine scientists, has developed a position statement on aversive stimuli in horse training [119].

##### Bits

A bit is a piece of tack—typically a slim bar of metal, rubber or plastic—that is placed into the mouth of an equid and used for communication and control. Bits are designed to rest in the natural diastema between the incisors and premolars and function by applying pressure to a number of different points in the mouth or head via the reins, cheekpieces or noseband, depending on the design of bit and bridle, of which there are many. Clearly, the horse’s mouth did not evolve to accommodate a bit, and bits can create pressure or pain on the bars, tongue or palate [77] and, in combination with the noseband, can pinch the buccal mucosa, tongue or lips.

Despite bits having been used for thousands of years as the primary means of control during riding and driving, it is often assumed they are both essential and ethical; however, research indicates that they can cause discomfort, pain, oral lesions, behavioural inhibition and, thus, also create fear and distress [188,191]. Any bit can create poor welfare if used incorrectly such as with excessive pressure, or by inexperienced riders/drivers [187,200]. However, risks are magnified with ‘stronger’ types of bit, which exert greater pressure or more severe action on the animal and, therefore, have more capacity to inflict pain and injury [77]. Mellor and Beausoleil [201] stated that “most horses exhibit clear behavioural evidence of aversion to a bit in their mouths, varying from the bit being a mild irritant to very painful”. In addition, certain types of bits may be associated with a reduction in the swallowing frequency of horses [202], and there is also evidence that bitted bridles can cause breathlessness, resulting in negative welfare impacts on horses [201]. When horses wearing bitted bridles and bitless bridles were compared, those wearing bitted bridles exhibited more chewing, opening of the mouth, pawing the ground and tail swishing, and during long-reining, their heart rate and heart rate variability were higher [203]. The study also found that horses wearing bitless bridles exhibited more head lowering during long-reining compared to those in the bitted bridles, which suggests that horses wearing bitless bridles performed at least as well as, if not better than, those in bitted bridles [203].

##### Spurs

Spurs are metal extensions attached to the heels of a rider’s boots to amplify or concentrate the pressure applied to the horse’s sides by the rider’s leg in order to transmit signals to encourage the horse to increase speed, increase activity or move in a specific direction. Forceful use of spurs has been found to cause hairs to be pulled out or broken off, resulting in worn or denuded areas on the horse’s sides, skin abrasions or bleeding, and the risk of lesions/hair loss significantly increase with the spur length and with rotating designs of spurs compared to static ones [193,194]. Lemon et al. [193] found that riders within competitive non-FEI disciplines were 1.53 times more likely to use spurs than recreational riders and 1.48 times more likely to use spurs than those competing in FEI disciplines. Acknowledging the potential detrimental impacts of spurs, British Showjumping has established a ruling for their use, effective from 1 January 2020, that states that the misuse of spurs is an offence and includes the (non-exhaustive) following: “spurs of excessively severe design are not to be worn and only listed spurs being acceptable; spurs must be of smooth material; the overall length of the spur is not to exceed 4 cm” [204].

##### Nosebands

A noseband is the part of the bridle that encircles the nose and jaw of the horse, of which there are many different designs. Nosebands are common in equestrian sport and are used to limit the extent to which horses can open their mouths to increase control [205,206]. The ISES defines a restrictive noseband as one that is tight enough to prevent the placement of two adult fingers between the noseband and the frontal nasal plane [205]. The pressure applied by restrictive nosebands cannot be released by the rider/driver during use, and therefore, these do not enable negative reinforcement principles to be applied (i.e., removal of an aversive stimulus when the animal performs the desired behaviour), which reflects learning theory [207]. This can compromise equine welfare by causing discomfort, pain or tissue damage; reducing the horse’s ability to swallow, yawn, chew and lick and potentially masking pain and discomfort [74,205,207,208]. Again, the ISES has developed a position statement to highlight the problems associated with restrictive nosebands and create awareness of the scientific evidence of potential adverse effects of their use on horses [205].

One study found that almost half of eventing and dressage competitors assessed in Ireland, England and Belgium used nosebands tightened to such an extent no fingers could be inserted beneath them [74]. This was contrary to the general recommendation that two adult human fingers should be able to fit under a fastened noseband, but surprisingly, noseband tightness levels are generally not regulated in competition [74]. It is interesting to note that regulations within some equestrian disciplines prohibit certain noseband types while others, such as elite dressage competitions, mandate types of nosebands that allow an increased tightness to be achieved (e.g., double bridles with cavesson nosebands, most commonly the crank type, which allows a doubling of the tightness achievable for a given amount of handler tightening effort) [74].

##### Whips

A riding whip, crop or stick usually consists of a long shaft of plastic, fiberglass or cane that is covered in leather, fabric, rubber or a similar material. Different designs of whips are used for different purposes and equestrian disciplines. Similar to spurs, whips are typically used as communication aids to amplify or concentrate signals or encourage the horse to increase speed, increase activity or move in a specific direction. From another perspective, as they provide an aversive stimulus, whips can be seen as tools for punishment, and their use as such can occur in horse training if the force of the whip does not diminish in response to the animal’s behavioural response, i.e., it is not used as negative reinforcement [77]. The appropriate use of negative reinforcement for training should involve a reduction in the force or application of the whip as the animal reacts to the stimulus [186].

The misuse of whips can cause pain, fear and distress both directly and also by making horses attempt to exercise beyond their capabilities [186], and excessive whip use may cause swelling, bruising or bleeding [194].

The use of whips in horse-racing has been much debated and has resulted in revision of the rules for whip use in British racing [209]. It is stipulated that the use of the whip is restricted to “safety, correction and encouragement”, and use of the whip to coerce is not permitted [209]. Whilst this seems commendable, and efforts to improve equine welfare in sport are to be encouraged, an ethical case could be made for prohibiting the use of whips in racing altogether. A detailed analysis of the review of whip use in horse-racing found no evidence was provided to support the conclusions that whip use is not painful, that whip use is not a welfare problem or that whip use is necessary for safety and encouragement, and failed to provide adequate justification for the continued use of the whip in horse-racing [210]. The analysis of the review also raised concerns about the review process, primarily that it was undertaken by the industry body responsible for promoting horse-racing, leading to potential conflicts of interest. The FEI’s General Regulations [211] state that abuse of a horse includes “to whip or beat a horse excessively”, unfortunately it does not define the term “excessively”. The Horse Welfare Board considered the issue of whip use in racing and determined that the number of whip offences, under the current rules, remains too high and suggest that the current sanctions are not an adequate deterrent [39]. Thus, there is still a need to evaluate the use of whips in all equestrian disciplines to effectively address whip usage and equine welfare [212].

##### Tongue-Ties

A tongue-tie is a strap, usually of rubber, leather or nylon stocking, that loops around the tongue to immobilise it by attaching it to the lower jaw or, sometimes, to the bit in the horse’s mouth and is primarily used for race-horses [213]. It is used to prevent a horse from placing the tongue over the bit, which can reduce the rider’s control, and may also be used in an attempt to prevent dorsal displacement of the soft palate [213,214]. Tongue-ties have been found to impair the airway in race-horses galloping at full speed [195,196], and it is also suggested that their use causes changes to both the behavioural and physiological parameters that are suggestive of a stress-related response [213]. There is also anecdotal evidence from people involved in the racing industry of the harm caused by tongue-ties, including horses being unable to eat for several days following the use of a tongue-tie during racing or training, physical damage to the tongue (e.g., lacerations), interrupted blood supply, tongue paralysis and permanent damage and tissue necrosis [196].

Acknowledging that equine tack and its use can compromise their welfare, the FEI’s Code of Conduct for the Welfare of the Horse states that “Tack must be designed and fitted to avoid the risk of pain or injury” and horses “must not be subjected to methods which are abusive or cause fear” [215]. However, evidence appears to indicate that tack continues to be a source of equine welfare compromise.

#### 3.3.2. Injuries and Death

In addition to injuries sustained from the use of equipment during training and competition, equine athletes are exposed to various other risks, which can result in acute and chronic injuries or even death [216]. Injury compromises equine welfare by inflicting discomfort or pain and may limit the ability to engage in positive behaviours, such as accessing food or playing with conspecifics. Rosanowski and colleagues [217], who assessed flat-racing events in Britain between 2000 and 2013 where horses required veterinary attention, reported a range of injuries and health conditions affecting race-horses. Soft tissue injuries other than tendon and ligament injuries were the most commonly occurring (24.1%), followed by gait observations (21.2%), respiratory conditions (21.2%) and then bone injuries (13.8%). There were 628 fatalities, with most (77.2%) the result of bone injuries, 64 due to cardiac conditions and 54 due to tendon and ligament injuries. Equine health programmes for preventable diseases have been successful in minimising losses (missed training or deaths) in the industry, and now, bone injuries are the principal reason for losses [218,219]. As a result of this influence, studies have investigated the risk factors in an effort to inform strategies to further reduce the rate of fatality in horses and enhancing horse welfare [220,221].

Between 2014 and 2017, a total of 683 horses died on British race-courses [121], and in 2018, there were 202 deaths, including seven at the four-day Cheltenham Festival alone [222]. In response to the level of equine fatality in racing, Bennet et al. [216] suggested that developing risk profiles for horses and riders could lead to interventions to reduce the number of falls, thus better protecting equid and human safety.

#### 3.3.3. Transportation

Equine athletes often compete at various locations, which typically requires them to undergo road transportation to and from different competition venues (usually in purpose-built horse boxes). The studies reviewed by Padalino [223] revealed that transportation causes physical and psychological distress in horses and can result in dehydration, injury, respiratory problems and gastrointestinal disease.

Transportation variables for equids include the duration of travel (behaviours relating to distress and balance increased during transport), direction of travel (forward-facing animals showed more balance-related behaviours than rear-facing animals), stall size (equids in narrower stall sizes showed more balance-related behaviours than those in wider stalls), experience (equids with less travelling experience showed increased signs of significant distress), temperament of the equid and driver ability/skill level [223,224]. However, it is challenging to assess the effects of transportation on equids due to confounding effects of the variables [223].

### 3.4. Life Stage: Subsequent to Participation in Competitive Sports

An equid’s sporting career generally comes to an end because of declining or poor performance, injury or aging, leading to retirement from competitive activities. In some cases, the animal’s owner may discontinue participation in sports due to injury or ill health, changing interests or a change in financial or personal circumstances. Equids who have undergone intensive sporting careers are susceptible to welfare compromises due to chronic injury or lameness (particularly related to hooves, limbs or spinal problems); respiratory conditions such as recurrent airway obstruction (‘broken wind’), which is especially common in former race-horses, or gastrointestinal problems associated with equine gastric ulceration syndrome (EGUS) [225]. EGUS aetiology is not yet fully understood but appears to be associated with the duration and intensity of exercise and a high-concentrate diet—both of which are pertinent to equids involved in sports.

The fate of equids post-competition varies but generally falls into one of the options below:

- Retirement: In some cases, sporting equids may be provided opportunity to enjoy a period of retirement or are retained as companions for other animals.
- Retraining: They may undergo training for alternative and less physically demanding activities (e.g., sold to a riding school or used for light hacking).
- Breeding: Depending on their value, breed and bloodlines, they may be used for breeding purposes, either small-scale or commercially.
- Meat/butchery: Equids of little economical value may be sold into the meat trade for slaughter and consumption (human or animal food products) or production of other by-products.
- Abandonment or neglect: If the owners are no longer willing or able to care for them, equids may be subject to abandonment or neglect or, alternatively, voluntarily surrendered to a rescue or rehoming charity.
- Euthanasia: Euthanasia may be warranted in cases of significant injury or intractable pain or occur at the behest of owners who no longer wish to retain the animal or pursue any of the alternative routes above. In the opinion of equid experts, one of the most important issues for horse welfare arises when old or ill horses are not promptly euthanised [69].

Behavioural problems can potentially arise because of a change in environment, management routine or through frustration or boredom, and abnormal behaviours, such as stereotypies, which may have formed during the equid’s competitive career, can also persist thereafter. Managing chronic physical or psychological challenges in a way that limits the extent of welfare compromise requires knowledgeable and sensitive ownership and a willingness and ability to adapt to the animal’s individual needs. However, with this provision and care, equids have the potential to enjoy a good quality of life throughout the post-competition phase.

The challenge of finding suitable homes for unwanted sports horses—or resorting to euthanasia/slaughter, in some cases—is particularly acute within the racing industry, possibly due to the scale of wastage, intensity of physical demands upon the animals and very short time span or sporting career, as race-horses often discontinue competitive racing whilst still relatively young, as their performance peaks around 4 to 5 years [226]. Accordingly, there are several UK charities dedicated solely to trying to address this issue, including New Beginnings, the Thoroughbred Rehabilitation Centre, the Racehorse Sanctuary and Retraining of Racehorses, which is funded by the British Horseracing Authority.

## 4. Institutions and Social Processes Influencing Equine Welfare (Ethics, Attitudes and Values)

Institutions are systems that facilitate decision-making and help to manage aspects of society and are seen as being both enabling by guiding people and constraining through the established rules [227]. When applied to equestrian sports, the relevant institutional components comprise the rules of competition, the equids, attributes of the stakeholders and the space where participants strive to achieve a common purpose [228]. This section endeavours to untangle the most significant elements that create barriers or provide opportunities for equine welfare improvement.

### 4.1. Established Rules for Informing about Equine Welfare

The rules, from an institutional perspective, relating to equine welfare are both formal and informal and are sources of information employed to promote good equine welfare in the industry. They include a broad range of printed and digital resources from government-imposed rules such as national legislation to self-imposed rules such as policies and guidelines from various governing bodies. Additionally, there are many and varied undocumented ways of information sharing based on traditions and customs. The legislation includes the *Animal Welfare Act 2006 (England and Wales)*, *Animal Health and Welfare (Scotland) Act 2006* and the *Welfare of Animals Act (Northern Ireland) 2011*. These acts primarily cater for domesticated and captive animals and apply to anyone who is responsible for their care. The relevant governments also produce codes of practice for how people should look after their animals to meet their needs under the respective acts and to provide practical advice, and each of the UK’s nations has produced codes of practice for the welfare of equids [229,230,231,232].

Specific to equestrians, British Equestrian, the national governing body for horse sports in the UK, has a webpage dedicated to equine welfare with a link to the FEI (the international governing body of equestrian sports) Code of Conduct for the Welfare of the Horse, to which it subscribes, as do their other member bodies. Similarly, the Hurlingham Polo Association, the governing body for polo in the UK, has the Polo Pony Welfare Booklet For Club Officials, Players and Grooms [233]. There are also equine welfare-specific pages on the websites of four member bodies: British Dressage, UK Polocrosse, British Horse Society and the Pony Club.

The British Horseracing Authority (BHA), responsible for the governance, administration and regulation of horse-racing and the wider horse-racing industry in Britain, has established an independently chaired Horse Welfare Board that, in February 2020, produced a five-year strategy with the following vision: “Respect for the horse is at the heart of everything we do: Every horse bred for racing will enjoy a life well lived” and with an outcome of “Collective lifetime responsibility—incorporating e.g., traceability across the lifetimes of horses bred for racing” [39]. The BHA also has webpages and an information sheet dedicated to equine welfare [234]. In addition, the BHA has developed rules of racing and supporting guidance, including equine antidoping rules (identifying prohibited substances and processes for sampling of horses), codes for horse equipment (identifying items that are permitted/not permitted) and penalty guidelines including breaches of the rules for using whips. There is also a range of training programmes, including diplomas and apprenticeships, for equine sports for stable staff, jockeys/riders and trainers that cover equine welfare [235,236,237].

For veterinarians, the British Veterinary Association has produced an animal welfare strategy [40], and the Royal College for Veterinary Surgeons has a code of professional conduct for veterinary surgeons [238]. These documents address broad issues such as animal welfare assessment, ethics, legislation and much more but do not directly mention the welfare of equids in the UK. As mentioned in Section 3.1.4, farriery is highly regulated, and there are three organisations concerned with the governance and standards of practice, namely the Worshipful Company of Farriers, the Farriers Registration Council and the British Farriers and Blacksmiths Association.

The Royal Society for the Prevention of Cruelty to Animals (RSPCA), the world’s oldest and largest animal welfare charity, has a range of policies on animal welfare, including one specifically addressing horses and ponies. These mention the use of equipment by riders and training methods that cause distress or suffering [239].

Additionally, books and journals provide a vast source of information specific to equine health and welfare related to equestrian sports. The examples include the *Equine Veterinary Journal*, the *Journal of Equine Veterinary Science* and special editions focussed on equine welfare in journals such as *Animal Welfare* and *Animals*.

Obtaining information directly from speaking to other people is another hugely influential source of information on equine welfare, and for many, is a preferred source, with veterinarians being particularly important [240].

All these rules and resources appear to reflect recognition, throughout the equestrian sector and broader society, of the need to mitigate risk and improve equine welfare, as well as for organisations to be seen to be addressing these issues at a time when there is increased public interest in animal welfare. The sheer volume of rules developed to minimise poor welfare for animals and those specific to equids clearly demonstrates that there is a problem; hence, attempts are being made to address it. A fundamental flaw in many of the rules relating to equine welfare is that the terms are often too vague and subjective for them to be interpreted in a way that truly benefits equids. For example, the FEI General Regulations include an article to address the abuse of horses that states: “No person may abuse a Horse during an Event or at any other time. “Abuse” means an action or omission which causes or is likely to cause pain or unnecessary discomfort to a Horse, including, but not limited to: To whip or beat a Horse excessively; To use spurs excessively or persistently; To use any device or equipment which causes excessive pain to the Horse upon knocking down an obstacle”. What is necessary discomfort? What is the measure for beating/whipping a horse excessively? Who decides?

As described in this section, there has been progress in improving equine welfare in the industry, such as changes to the rules and structures of organised sports (for example, whipping limitations, the prohibition of ‘tubing’ and the establishment of the Horse Welfare Board by British Racing to produce a welfare strategy). On the surface, the production of multiple sources of information by a broad range of stakeholders using rousing language that suggests a real concern appears to be very positive, yet the equids used in sports are still exposed to significant welfare risks and actual harm, as discussed above. A potential reason for this is the reliance on rules that, at times, can be vague and vulnerable to subjective interpretation; hence, welfare compromise can still occur despite UK legislation stating that animals’ needs are met, including protection from “pain, suffering, injury and disease” (*Animal Welfare Act 2006*). Additionally, rules can be rendered ineffective due to weak and/or sporadic enforcement.

### 4.2. Ethics

From an ethical perspective, views on using animals for sports vary widely, from a moralistic stance that no sentient animals should ever be used by humans [241] and have inviolable rights [242] to a utilitarian view of accepting the use of animals when the benefits to humans outweighs the loss to the animals [243]. The key to the latter view is determining the relative values of benefit and loss. Alternatively, people might be drawn to using horses based on the physical appeal and beauty of the animals, or they might take a dominionistic view of mastery, physical control and dominance over animals [244]. To illustrate, the use of a bit, placing an object into the mouth of an equid and attaching it to reins to control her through pressure (and potentially pain) while the human sits on her back, is a clear example of control and dominance. Indeed, Morris [245] said, “If the dog is man’s best friend, then the horse could be well described as man’s best slave”.

Two polarising viewpoints on how to respond to the exploitation of animals commonly in use are (i) those of the welfarists/reformists who believe it is acceptable for humans to use animals so long as this done humanely, and who seek to improve the conditions for animals; and (ii) the perspective of the abolitionists, who work to eliminate all uses of animals that are considered to cause pain and suffering [241,242].

For some, the use of animals for competitive sport is less ethically justifiable than other uses of animals, because sport is considered as a trivial purpose [246]. It can also be argued that, as long as the animal is well looked after and has a life worth living, then it is ethically acceptable [246]; again, this depends on the definition of ‘well looked after’ and ‘a life worth living’ and the extent to which this is appraised from the animal’s perspective rather than through an anthropomorphic lens. The relationships formed between humans and equids can be seen as symbiotic if considering survival of the species, but the power dynamic within this relationship is not equal [247].

Many people believe that animals exist for human benefit and accept domestication and human-induced selection to ‘improve’ animals, or even think that humans have a moral obligation to ‘enhance’ other species with which we share the planet [248]. At the other end of the spectrum are those people who argue that domestication harms animals and is therefore a morally indefensible process [249]. Whilst the domestication process provides equids with some benefits, such as the provision of food, shelter and protection from predators, there are also costs, and these include the restriction of movement, social interaction, reproductive success and maternal behaviour [250] and, often, a lack of agency and free choice.

Additionally, there are the ethics of modifying or ‘enhancing’ horses through selective breeding for characteristics desirable to humans (e.g., speed, endurance or jumping ability) and the problems associated with inbreeding depression, as discussed in Section 3.2.1. This form of manipulation does not bestow demonstrable benefits to the animals involved, often compromising their welfare, and is undertaken to advantage their human owners or humans more broadly in the case of conserving particular breeds for future human enjoyment [251].

Another perspective considers animals as being engaged in work or labour, in this case equids as a non-consenting resource for sport, i.e., they are not co-workers with agency over their level of participation. In the case of using animals in sport, there is a unique motivation whereby some people are driven by the desire to win [77], perhaps due to the status or gratification, or in some cases the financial reward. In horse-racing, the athletic ability of the jockey, prepared to invest huge effort and self-sacrifice, is not necessarily shared by the horse. Additionally, as the British Horseracing Association [122] points out, “all elite sports and all activities involving horses, there is an element of risk”, but horses do not consent to accepting the risks from such activities. However, there is a view that some interspecies relations, combining robust rights with notions of shared membership and cooperation, may exist [252].

The broad range of perceptions, attitudes and values makes it particularly challenging for equestrian sports regulators to ensure harm is minimised and welfare optimised. The need for a set of moral principles relating to conduct regarding equestrian sports has been acknowledged but to date has been discipline specific or ad hoc [253]. The aim of such principles should be to provide a comprehensive and defensible method for identifying the ethically important aspects within the industry. To progress the issue and help stakeholders in equestrian sports consider the “inevitable ethical questions” that arise, Campbell [253] has developed the first theoretical framework to enable the stakeholders to “underwrite the continuation of the social license to use horses in sport and to enable those within equestrian sport to critically assess existing and proposed practices and to make welfare-improving adjustments to practice”. Fundamentally, the process defines the ethical issues, identifies the stakeholders and their interests, assesses the pertinent evidence, identifies relevant legislation/regulation, and then applies a harm–benefit analysis to the issue. It is proposed that the framework can operate at a national level or more locally, and across equestrian disciplines. It appears to have the potential to be an important tool and the results of testing and refining currently underway could be helpful for improving equid welfare.

### 4.3. Stakeholders

Now focusing on the stakeholders within the equine sports industry, including owners, veterinarians, trainers, stable staff and riders, it is clear there is a broad range of attitudes, values, perceptions and practices which can influence equine welfare. Veterinarians, for example, play a crucial role by providing preventative and remedial medical care for horses, and an important consideration is veterinary ethics. Veterinarians are regularly faced with ethical dilemmas with consequences for patients, clients, colleagues (e.g., differing opinions) and in some cases their own business/livelihood (e.g., stopping a horse from competing on account of injury when a trainer may wish to proceed with competition). At times the jobs of veterinarians within equestrian sports are all the more challenging when they are faced with potential ethical dilemmas from conflict between their duty of care for performance horses and their responsibilities to the owners/trainers/managers who are purchasing medical care and have an interest in keeping the equid competing [243]. Ideally, resolving such situations involves principles that underlie professional reasoning, and processes that govern the veterinarian’s intentions and actions, helping to make rational and defensible judgements, improve consistency, and enable them to be resilient, confident, behave better and be more effective [254,255]. Having said this, veterinarians have been found to value their own personal and collective experiences over research-based evidence and consensus [256].

There is a broad spectrum between harmful and acceptable welfare risks of equestrian sports with some sports always being higher risk than others, for example eventing and racing [216,257]. The acceptance of these risks is often determined by the attitude of the people involved and the training and management practices they employ, and potentially the rewards that may result from success (psychological, social or financial).

Equine sports differ from other uses of animals by providing a unique incentive of ‘winning’, “a human psychological satisfaction that goes beyond the notion of pleasure or even success because it requires that we out-perform other people” [186]. The urge/pressure for a horse to win can be high, leading many owners, trainers and riders to consider interventions (such as using whips, spurs or even performance-enhancing drugs) that they perceive may increase chances of winning or safety, or risk giving up the possibility of a first place [186]. The pressure may be particularly great in horse-racing where earning potential is huge and determines the value of racing Thoroughbreds [258], both at the time of competition and subsequently at stud. Trainers’ highly variable incomes that are bolstered by receiving a percentage of any prize money their horses might accrue, may be another reason for them to push their horses to perform exceptionally [259].

The use of illegal drugs for horses by unscrupulous people to improve their competitive performance or to mask management deficiencies that cause pain and disease (i.e., avoidable suffering), occurs within equine sports and this form of cheating is clearly unethical and often constitutes an abuse of the welfare of the horses involved [246]. Furthermore, some riders use drugs and alcohol for themselves, compromising their own safety as well as that of other riders and horses [260]. Evidently, these activities are not considered acceptable by some factions, hence rules and procedures exist to prohibit their use [209,261].

The racing industry has been identified as being particularly vulnerable to compromising horse welfare [192]. Bergmann [262] states that the Thoroughbred racing industry principally seeks to address only the “most egregious welfare violations on and off the track, to influence the public’s perception of the industry and its treatment of the thoroughbred; and to focus on productivity, efficiency and optimisation of the commodifiable characteristics of the thoroughbred”, which is unlikely to result in significant welfare improvements for the equids involved. Within the British horse-racing industry, staff shortages were perceived as directly affecting horse welfare, including the opportunity for staff to develop a human–horse relationship [259]. The same study also found perceptions that horse welfare was compromised as a result of poor employee relations due to a lack of recognition, communication and respect leading to negative employee attitudes, behaviour and staff retention.

Trainers, stable staff and riders have a duty of care and a legal requirement under UK animal welfare law to ensure appropriate health and welfare of the horses in their care, but their perceptions can result in a range of equine welfare problems including horses being under- or over-weight, lack of turn-out due to concern about injury, the inappropriate use of training aids and horses being kept in environments that may fail to meet all their psychological and behavioural needs [192]. Long-standing traditions and experiential learning are particularly prevalent in the racing industry [247] and appear to impede the integration of scientific knowledge [118,263]. A key challenge when discussing horse welfare is that it invokes strong feelings from all sides of debates on the subject with different perspectives based on perceptions and understanding, tradition, culture and/or desired outcomes. Those external to the industry may not have a detailed comprehension of the issues and pressures within it, and similarly, those within it (potentially all their lives) may not be well-placed to understand alternative perspectives and value judgements, or to critically evaluate the prevailing norms and traditions. It is therefore important to focus on objective evidence of welfare from the animals’ perspective—and for implementation strategies to be effective, the owners, carers, riders/drivers, trainers and managers of equids need to be motivated and open to change. Personality differences can be influential too, for example Wolframm et al. [264] found that competitive riders exhibited higher levels of extroversion and conscientiousness than recreational riders.

For some animal welfare proponents, the priority may be ensuring animals are safe from harm by providing for their physiological needs (e.g., shelter, clean water, sanitary living conditions) and physical health (e.g., being disease-free, having injuries treated and suitable nutrition)—these are the basic requirements for keeping animals alive. For others there will be a desire to strive for higher levels of welfare by catering for their behavioural and psychological needs, including social interaction, mental stimulation and providing agency and choice. This latter group may have honourable intentions but may be constrained by their understanding of what their charges really require, and their budgets, including both time and financial, limiting scope to implement all possible activities to achieve optimal welfare. Another consideration is that there will be huge variation of standards within and between all the different stakeholder groups and individuals. A fundamental challenge will be resolving conflicts between the interests of stakeholders, which often do not align, resulting in a need to identify which stakeholder’s interests should be given priority [253].

Both class and gender inequalities exist within horse-racing [265] and research has also exposed gender-based differences in equid-directed behaviours with men being 2.88 times more likely to use spurs than females, and having higher frequency of spur use overall (in training and competitions) compared with females [193].

### 4.4. Anthropomorphism and the Human-Horse Relationship

Anthropomorphism can also influence people’s perceptions of equids and may play a part in suboptimal equine welfare with misinterpretations of the responses of trained horses, such as the use of terms like “mad”, “lazy”, “keen”, and “stubborn” [186] or the use of the word “naughty”, that inappropriately shifts the blame from the rider to the horse [77]. It can be problematic using an anthropomorphic framework to explain rider–horse interactions which can disguise and justify abuse of horses who offer undesirable responses [118]. Anthropomorphism is deemed to be “unhelpful at best and may promote poor welfare at worst, particularly when it comes to describing problem behaviours as having some malevolent component” [77]. It is worth noting that anthropomorphism can be useful for developing a greater understanding of human–horse relationships and assisting humans to develop some empathy and insight into their horse’s experiences [266]. Some riders hold strong bonds with their horses, but these interactions are generally instigated by humans for the benefit of humans and interpreted through human perceptions and values [267].

Equids are of cultural importance in the UK having played significant roles in human history, and these relationships and interactions have varied over time [268]. There has been a significant shift in the human-horse relationship from a utility-based role in agriculture, transport and warfare, to one more focussed on sport, leisure and tourism [266]. Today, there is a cohort of people who interact with horses who acknowledge friendships with them and perceive equids as providing “companionship, security and warmth” [267]. Many people also admire the aesthetic appeal of horses on account of their beauty, grace and speed [269], as evidenced by participation in competitive showing events or classes in which animals are judged based on demonstration of desirable breed-specific physical traits.

### 4.5. Science Communication

A further barrier to addressing equine welfare issues relates to the effective communication of, and engagement with the scientific evidence, particularly with non-experts. This is especially problematic when it comes to addressing the complexities of animal welfare, as described above. There is also the challenge of trust in an era when people increasingly question scientific results and expertise as an outcome of those communicating being tainted by special interests or doubts raised by the information on social media [270]. It is difficult for scientists wishing to disseminate justice-centred information while having to deal with emotion, politics, unbalanced power dynamics and conflict [271].

As demonstrated above, it is not simply an empirical issue using objective measures of animal welfare, the human social influences will lead to different welfare emphases and different outcomes [272]. The final, and possibly greatest, challenge is implementing a process that incorporates all the social complexities identified above.

## 5. Conclusions

Evidently, there are very many welfare risks to which equids used in sports continue to be exposed. It is also evident that significant improvements have occurred in recent times, but there remain barriers to reducing the risks to an acceptable level. This is clearly not helped by weak institutions which may result from poorly defined terms either intentionally or not, and due to the complexity of issues and/or differing opinions. The plethora of policies, regulation and research papers covering equine welfare is not yet enough to address significant problems that still occur. Of course, there are many involved in the industry who develop deep emotional relationships with horses and truly care about them, but there remain cultures and practices that push the boundaries, resulting in poor welfare before, during and after their competitive career, as explored above. Additionally, conclusions drawn from scientific evidence should not be ignored and overridden on the basis of the large number of people who participate in the sport or the large sums generated. Comparisons could be made with the climate change debate or even the tobacco industry debacle, where the evidence exists but the human elements, including perceptions, attitudes, values, power and connections of individuals, overly influence the debate, leading to little or slow change. A fundamental problem is that stakeholders, from scientists to managers to policymakers, all have different values, approaches, interests, and goals and therefore come up with many and varied answers to the problems. Scientific evidence demonstrates that equids are suffering for sports and, therefore, there is a need to build on evolving new processes that incorporate animal welfare science together with human behaviour change strategies.

What is critical for implementing change is not a provision of a list of dos and do nots but a robust framework that incorporates the following:

Process

- Acknowledge that there are currently risks of poor welfare at multiple stages within the industry and be prepared to invest both time and other resources for the long term to eradicate poor welfare and promote positive welfare experiences for sports equids.
- Ensure there is long-term investment in research to identify welfare risks to equids used for sports.
- Implement formal, objective assessment and change processes, overseen by truly independent bodies of experts to identify, prioritise and implement changes to improve poor welfare.
- Produce implementation plans with clearly identified outputs that address prioritised welfare risks.
- Implement management actions.
- Monitor outputs.
- Evaluate welfare outcomes for equids.
- Report on welfare outcomes, sharing knowledge throughout the equine sports sector.
- If required, refine actions, and re-implement them.

Stakeholders

- Involve all stakeholders from a broad range of relevant professions and experiences including industry, community, research, charity and government sectors.
- Include natural scientists (including from the multi-disciplinary field of animal welfare science), social scientists, psychologists, and economists to ensure related but highly important/relevant issues are incorporated.

Knowledge and practice

- Include structured reviews of relevant literature (including and beyond all of the different contributors to equine welfare addressed in this review) to facilitate learning and inform change processes to ensure work goes beyond the backgrounds, experiences and biases of the individuals involved.
- Identify all welfare risks and causative factors.
- Where there is epistemic uncertainty, utilise structured elicitation of expert judgement to inform decision-making (e.g., via the Delphi method) to reduce error and bias among experts including overconfidence, anchoring to the available data and definitional ambiguity.
- Incorporate adequate flexibility within equine management processes to, for example, address individual differences of equids, such as previous learning, personality and personal preferences.
- Incorporate the precautionary principle to take preventive action in the face of uncertainty.

This review has focused on equestrian sports within the UK, but the identified welfare risks and recommendations elaborated above could also be useful for practitioners and stakeholders working on the welfare of equids used for sports elsewhere in the world, or the welfare of equids involved in other activities, such as leisure, work or at stud.

Fundamentally, there is a need to align the theory and evidence base on animal welfare and equine science with what is actually happening to equids in practice. There is a need for a formal process to improve welfare in equestrian sports, and such a process needs to assess equine welfare, identify the risk factors, prioritise the risk factors (based on the severity, intensity and duration of poor welfare) and implement targeted welfare-improvement interventions (including education of relevant stakeholders in the field of equestrian sports) that focus on the most severe issues causing the greatest amount of suffering. In some cases, it will also be necessary to conduct further research to better understand risk factors for poor welfare, opportunities for positive welfare, and efficacy of welfare improvement interventions—something that should be welcomed, encouraged and supported by the equestrian sports industry.
